# Notes and News

**Published:** 1899-07-29

**Authors:** 


### Notes and News.

(Collected and Communicated.)

In the Queen's Bench Division of the High Court of Justice application was made last week by the Local Government Board for a mandamus commanding the Guardians of the poor of the parish of Leicester to appoint a vaccination officer. The Court granted a rule *nisi*, making it returnable this week, and a copy of the order was served upon the members of the board of guardians. In consequence of this a special meeting of the guardians was held last Friday to consider their course of action, and it was decided with one dissentient to instruct counsel to show cause against the issue of the mandamus. And so the matter stands. In any case the prospect is disheartening. If the Local Government Board wins the day, we have to look forward to a series of theatrical martyrdoms, and to constant agitation against the Act; while if the guardians win the Act will become a dead letter over large areas. So much for the intrusion of politics into health legislation.

It appears that out of the seven objecting Guardians at Leicester to the election of a vaccination officer six are women. It is very unfortunate for the progress of the women's movement that a retrograde policy should so soon have declared itself amongst the comparatively few who hold office.

The League of Mercy is making good progress although a very large section of those who will, it is hoped, in future co-operate in the beneficent scheme as yet know nothing, or very little, about it. The fact that the presentation of the certificates to the nurses of the Royal National Pension Fund was chosen, also, as an opportunity for the Prince to identify himself closely with the workers on behalf of his Hospital Fund for London, will do much to stimulate individual interest throughout the various districts in London. On this occasion the presidents and lady presidents already elected were invited to view the ceremony of the presentation of the certificates to the nurses, and were afterwards received by the Prince and Princess. The League of Mercy is worked very much in the same way as the Primrose League. Districts are presided over by presidents and lady presidents, and these, in their term, associate vice-presidents with them, whose duty it is to collect a minimum number of 20 members each. The members undertake to collect at least 20 subscribers of at least one shilling each for the League. In this way a well-regulated sort of snowball is set up, and interest in the hospitals is everywhere stimulated. All information can be obtained from the office of the Fund, at 28, Southampton Street, Strand.

A novel conference is to take place in Liverpool this week. This is a conference of deaf mutes. Delegates have arrived from all parts of the kingdom, and papers will be given upon all subjects furthering the education of persons similarly afflicted.

At the council meeting of the Metropolitan Hospital Sunday Fund, which is being held whilst we go to press, the important announcement was made that the total amount collected by its agency for the London hospitals since its foundation now reaches upwards of £1,000,000. This is a truly remarkable and splendid result.

Andrew Duncan, Esq., M.D., F.R.C.S., and Oswald Baker, Esq., L.R.C.S., L.R.C.P., have been elected respectively physician to in-patients and physician to out-patients of the Seamen's Hospital Society, with duties at the branch hospital in the Royal Victoria and Albert Docks, in connection with the London School of Tropical Medicine.

There seems to be a little lack of harmony between the Council of the British Medical Association and the Parliamentary Bills Committee. For a long time back the latter body, if not all-powerful, has at least been the leading committee, the one which has been most expressive of the aims of the association. Things now are rather different, and the Council has referred back the recommendations of the committee in regard to amendment of the Medical Acts for further consideration. It is stated, indeed, that it is the deliberate opinion of the Council that in regard to three points —namely, the reconstitution of the General Medical Council, the one portal system, and increased representation on the General Medical Council—the proposals of the Parliamentary Bills Committee are unworkable. Clearly reaction is to be scented in the air, and the Council is asserting itself.

The question of reform in the Royal College of Surgeons is being somewhat heatedly discussed. The Council is evidently determined not to introduce into the proposed new charter anything beyond the institution of honorary fellowship, while those who have for years been asking for reform not unnaturally look on the occurrence of the centenary, and the desire of the Council to celebrate the event by conferring honorary fellowships, as the best conceivable opportunity for bringing their demands to a practical issue. They maintain that the most becoming manner in which the centenary could possibly be celebrated would be by the admission of the members of the college to a larger share in its government; and when it is objected that this cannot be done without an alteration of the charter, they answer at once, "What better opportunity, then, could possibly occur than the present, when the Council is actually on the point of getting a new charter for other purposes?" The Council indeed, like a railway porter, is trundling an enormous barrow, its "New Charter," just for the sake of carrying a little hand-bag, its "Honorary Fellowship." But when the Discontented Member, struggling along with his bundle of parcels loosely tied together with a bit of "Reform" string says, "Just put these on your barrow, that's a good fellow, there's plenty of room," the porter does not see it. Selfish man! So hard words fly around, and the Discontented Member threatens dreadful things—even, we believe, to upset the barrow.

